# Development and validation of the Value-Expectancy STEM Assessment Scale for students in higher education

**DOI:** 10.1186/s40594-018-0121-8

**Published:** 2018-06-15

**Authors:** Joseph Appianing, Richard N. Van Eck

**Affiliations:** 1 Center for Instructional and Learning Technologies, University of North Dakota, Grand Forks, ND USA; 2 Associate Dean for Teaching and Learning at the School of Medicine and Health Sciences, University of North Dakota, Grand Forks, ND USA

**Keywords:** Women and STEM, STEM education, STEM, Motivation, Expectancy-value theory, Value Interest and Expectation Scale, VIES

## Abstract

**Background:**

Science, technology, engineering, and math (STEM) jobs are expected to make up a significant portion of the U.S. workforce. Unfortunately, the trend in retaining students in STEM majors has been going down. If higher education institutions are going to retain more students in STEM majors, it will be important to understand who leaves STEM fields and why. More than 32% of women college students who declare a STEM major are likely to switch to non-STEM majors before they graduate, whereas only 25% of their male counterparts do so, and women may be as much as 1.5 times more likely than men to leave STEM fields. Thus, women represent a significant potential source for increasing STEM majors. Research suggests that values and expectations are powerful predictors of motivation and persistence in a wide variety of activities, tasks, and careers. This paper describes the development and validation of an instrument to measure student motivation, particularly that of women, leading to decisions to persist in or switch out of collegiate STEM programs.

**Results:**

The Value-Expectancy STEM Assessment Scale (VESAS), adapted from the Values, Interest, and Expectations Scale, or VIES, was validated with 356 women students from a Midwestern research university as part of a larger study on the reasons that women persist or leave STEM majors. A confirmatory factor analysis suggested a two-factor model, which reflected the components of Eccles et al.’s expectancy-value model. Cronbach’s alphas suggested that the VESAS subscales had high internal consistency. Statistically significant differences were found between STEM *switchers* and *persisters* on all of the VESAS subscales, thus lending additional support for the validity of the instrument.

**Conclusions:**

The VESAS appears to be a valid scale for measuring female college students’ value for and expectations regarding STEM majors. Suggestions are made for use of the VESAS in future studies to examine how motivations of women students enrolled in STEM programs change over time and to better understand when retention interventions might be needed and with whom.

## Statement of the problem

Science, technology, engineering, and math (STEM) fields are considered the drivers of innovation in the United States (U.S.) economy (Olson and Riordan 2012; Palmer and Wood 2013), and STEM jobs are expected to make up a significant portion of the U.S. workforce (Chen 2013; Ellis et al. 2016; Olson and Riordan 2012). The President’s Council of Advisors on Science and Technology (Olson and Riordan 2012) report suggests that academic institutions in the USA will need to increase the number of STEM graduates by one million over the next decade in order to meet the demand. Increasing the retention rate of STEM majors in the “STEM pipeline” (Soe and Yakura 2008) by even a small percentage would be a fast, cost-efficient way to produce the additional STEM graduates required for the U.S. workplace (Chen 2013; Ehrenberg 2010; Ellis et al. 2016; Olson and Riordan 2012).

Unfortunately, many students in the USA leave collegiate STEM programs after declaring their intention to major in STEM fields. For instance, Wilson et al. (2012) noted that, generally, less than half of the first-year students who declare STEM majors in the USA go on to graduate with STEM degrees. This finding supports the work of Chen (2013), whose 6-year longitudinal study of beginning postsecondary students in the USA found that of the approximately 50% of students who left their STEM fields, about half switched to non-STEM fields and the remaining half dropped out of college without earning any degree.

While the empirical research regarding the switching rates among students across different academic majors is limited, Chen’s (2013) research is worth noting. Findings from her 6-year longitudinal study among students majoring in humanities and education, for example, show that about 56–62% switched majors as compared to those in STEM fields (48%). This suggests that major switching in STEM is actually lower than in other fields. Chen further notes that the attrition rates for students majoring in business and social sciences were 50 and 45%, respectively, which were comparable to those in STEM fields.

Astorne-Figari and Speer (2017) point out that women are nearly 75% more likely to switch out of STEM majors than their male counterparts irrespective of performance (grades). In validating their model of major switching using the National Longitudinal Survey of Youth 1997 (a nationally representative dataset of 8984 youth born between 1980 and 1984), they found that the switch-out rates for STEM overall are similar to those of other majors, that being female was not a significant predictor of switching out of a first major, that *competitiveness* of the major was a larger predictor of switching than GPA or gender, and that women switch out of competitive majors at twice the rate of males. Given these data, it would seem that women are no more likely to switch majors than men are and that they tend to switch out of competitive majors (e.g., STEM majors) more often than men for reasons other than grades.

If higher education institutions are going to retain more students in STEM majors, it will be important to understand who leaves STEM fields and why. The answers to both questions are highly varied and continue to be the subject of intense research. Research has led to some consensus that women and non-Asian minority students leave STEM fields at higher rates than their counterparts (Chen 2015), as do first-generation students^1^ and those from low-income backgrounds (Shaw and Barbuti 2010). More than 32% of women college students who declare a STEM major are likely to switch to non-STEM majors before they graduate, whereas only 25% of their male counterparts do so (Davignon 2016). Other research has shown that women may be as much as 1.5 times more likely than men to leave STEM fields (Ellis et al. 2016).

Why do women and other underrepresented groups leave STEM fields at higher rates than other groups? Previous research indicates that there are various reasons, including poor grades and performance in STEM classes compared to non-STEM classes (Ost 2010; Rask 2010), loss of interest in STEM majors (Johnson 2012), growing interest in non-STEM majors (Watkins and Mazur 2013), lack of role models and mentors (Drury et al. 2011), feelings of isolation because too few peers pursue STEM majors (Chen 2013), and discrimination on the basis of sex, race, or ethnicity in STEM education and the workplace (Hill et al. 2010). Many of these reasons are interrelated, creating difficulty in developing a clear picture of the situation.

Because more than half of students who leave collegiate STEM programs are women, scholars have focused their efforts on understanding why women leave STEM fields at a higher rate than men. Some scholars argue that the underrepresentation of women in STEM fields is attributable to attitude rather than to aptitude (e.g., Chen 2013; Ehrenberg 2010; Else-Quest et al. 2013; Palmer and Wood 2013), citing research that shows women who leave STEM fields are often just as capable as those who remain in STEM fields (Chen 2015; Hill et al. 2010). Other scholars argue that the underrepresentation of women in STEM fields is linked to the value that they attach to, and their expectations for success in, STEM fields and careers. Research suggests that values and expectations are powerful predictors of motivation and persistence in a wide variety of activities, tasks, and careers (Eccles 2007, 2011a, 2011b; Wigfield 1994). The expectancy-value model, developed by Eccles, Wigfield, and their colleagues (Eccles 1994, 2005; Eccles et al. 1983; Wigfield 1994; Wigfield and Eccles 1992, 2000), may be among the most important models for explaining the decision to seek, persist in, or leave STEM majors. The purpose of this study was to develop and validate a scale to measure the values and expectations of women as they relate to their decision to seek, persist, or switch out of STEM majors.

## Theoretical framework

### The expectancy-value model of achievement motivation

Research has shown that men and women differ in terms of their educational and vocational choices (Eccles 1994). In STEM fields, for instance, the proportion of women in the medical, biological, and social sciences (especially psychology) is relatively high, whereas in disciplines such as physical sciences, engineering, and computer sciences, women are underrepresented as compared to men (Eccles 2011b; Rask 2010). The basic premise of the expectancy-value model is that choice of achievement-related tasks, performance, and persistence are most directly predicted by one’s expectations for success on those tasks and the extent to which one values the tasks (Eccles 2007, 2011a, 2011b; Wigfield 1994).

An individual’s expectations for success and subjective task value are shaped over time by several personal and cultural/social influences (Eccles 2007, 2011a, 2011b; Wigfield 1994). Influences from one’s cultural/social milieu may include social role and cultural stereotypes of subject matter and occupational characteristics, an individual’s gender, the beliefs and behaviors of socializers (e.g., parents and teachers), and the abilities and previous achievement-related experiences of the individuals (Eccles et al. 1983). Personal influences may include ability beliefs, socializer beliefs, expectations, social roles and stereotypes, the perceived difficulty of the related tasks, one’s goals, self-schemata, and affective memories, as well as one’s interpretations of one’s own previous achievement-related experiences and the various social influences (Eccles 2007, 2011a, 2011b; Wigfield 1994).

The expectations for success and subjective task value are presumed to directly influence achievement-related tasks, performance, and persistence (Wigfield and Eccles 2000). When expectations for success and value of STEM fields and majors are high, the person is much more likely to seek, persist in, and graduate from STEM majors. The opposite is true when both factors are low. However, these factors are not always aligned. One can have low perceived value for STEM but high expectations for success, and vice versa. The interaction of these factors is expected to have differing effects on decisions to seek out, persist in, and graduate from STEM majors and is thus important to researchers seeking to understand the reasons some women do not choose STEM majors or choose to leave STEM majors.

#### Expectations for success beliefs

Wigfield and Eccles (2000) define expectations for success as one’s beliefs about how well one expects to perform on a given task. Although there are many factors that influence expectations for success, self-efficacy is one of the most important because it has been linked to expectancies, performance, and persistence in achievement-related tasks (Eccles 2005; Wigfield 1994; Wigfield and Eccles 1992). Achievement-related tasks include the decision to choose and persist in STEM majors.

*Self-efficacy beliefs* refer to the beliefs that individuals hold about their abilities to successfully execute those activities necessary to achieve desired outcomes (Bandura 1977; Hutchison et al. 2006). An individual who has high self-efficacy would be more willing to engage, work harder, and persist longer in the face of failure, challenges, and difficulties than an individual who doubts his own abilities (Renninger and Hidi 2016). Self-efficacy is associated with the anticipated level of attainment required and with the strength of one’s belief that that level of attainment is achievable. As such, it differs from self-confidence, which refers only to the *strength* of a belief in one’s ability.

Research has linked self-efficacy beliefs of students to their level of interest (Bong et al. 2015; Lent et al. 2003), performance (Hackett et al. 1992; Pajares 1996), and persistence (Lent et al. 1984; Multon et al. 1991) in college majors. Research has shown that, compared to men, women have lower self-efficacy beliefs in STEM majors (Backer and Halualani 2012), particularly mathematics, chemistry, engineering, and computer science majors (Beyer 2014; Louis and Mistele 2012). Studies also indicate that women who switch collegiate STEM majors (switchers) have lower STEM self-efficacy than women who persist in STEM majors (persisters), even though they may have equal or better levels of academic achievement (Backer and Halualani 2012; Hutchison et al. 2006).

The literature identifies four primary influences from which self-efficacy beliefs are formed: mastery experience, vicarious experience, social persuasion, and physiological reaction (Hutchison et al. 2006; Plunkett et al. 2010; Rittmayer and Beier 2009; Zimmerman 2000). *Mastery experience* has to do with the individual’s previous success (or failure) at performing a given task. *Vicarious experience* refers to the knowledge and confidence that an individual gains by observing others (e.g., role models) perform a task in a certain area or field (Hutchison et al. 2006; Rittmayer and Beier 2009). *Social persuasion* refers to the feedback, encouragement, and support that a person receives from others, especially significant others such as parents and teachers (Plunkett et al. 2010; Rittmayer and Beier 2009). Finally, *physiological reaction* relates to physical reactions and emotions (e.g., fear of failure, fatigue, stress, anxiety, and nervousness) that an individual experiences during the execution of a task (Hutchison et al. 2006; Plunkett et al. 2010; Rittmayer and Beier 2009; Zimmerman 2000).

#### Subjective task value beliefs

Eccles (2005) describes task value as “… a quality of the task that contributes to the increasing or decreasing probability that an individual will select it” (p. 109) and further defines the quality of the task in terms of four components: (1) attainment value, (2) intrinsic (interest) value, (3) utility value, and (4) cost value.

The *attainment value* is defined as the personal importance individuals attach to doing well on a given task (Eccles 2005; Wigfield and Eccles 2000)—in other words, how well the given task fits the individual’s self-identity. A woman from a well-respected family known in the community as “a family of medical doctors” who is currently enrolled in a premed major at her university would be expected to have a high attainment value for the said major because doing well in this major might affirm her personal or social identity (Eccles 2005; Eccles et al. 1983).

*Intrinsic (interest) value* has to do with the inherent, immediate enjoyment that an individual gains from engaging in a given task or the anticipated enjoyment that individual expects to get while engaging in the task (Eccles 2005; Eccles et al. 1983).

The *utility value* refers to the usefulness of the task or how a given task fits into the future goals/plans of an individual, for example career goals (Eccles 2005; Wigfield and Eccles 1992, 2000). A student aspiring to be a medical doctor may dislike chemistry classes, yet because all premed students are required to take a chemistry course, and the instrumentality of chemistry helps achieve the aspirational goal, which, in turn, imparts a high value to the course (Eccles et al. 1983).

The *relative cost* refers to what an individual would have to give up in order to engage in a given task (Eccles 2005; Wigfield 1994), and may include the effort required to succeed, the time lost for other valued tasks, and the psychological cost of failure or emotional trauma resulting from failure (Eccles et al. 1983). Students must choose between various tasks, such as forgoing a movie in order to do a biology assignment. The relative cost is the movie the student did not attend.

Being able to measure these constructs and attitudes is of critical importance to advancing research in how to retain more women in the STEM majors they choose. Within the past decade, researchers have developed valid and reliable instruments to measure students’ attitudes toward STEM programs and careers. However, these instruments focused on middle and high school students’ attitudes toward STEM programs and careers rather than on those of college students (e.g., Guzey et al. 2014; Mahoney 2010; Tyler-Wood et al. 2010; Unfried et al. 2015). Furthermore, none of the instruments reported here were based specifically on Eccles et al.’s (1983) expectancy-value model.

In 2015, the authors developed and validated the Value, Interest, and Expectancy Scale (VIES) to measure the motivational aspects of men and women who pursue information computing technology (ICT) careers (Appianing and Van Eck 2015). Because the researchers sought to next study the reasons that women in particular do not pursue or persist in STEM majors, a new scale was subsequently developed based on the VIES with only minor changes to the wording of the scale (e.g., to replace ICT with STEM). This new scale, the Value-Expectancy STEM Assessment Scale (VESAS), was validated as part of a larger study of the reasons that women seek, persist in, or switch out of STEM majors in college. As the purpose of the current article is to describe the validation of the VESAS, details relating to the other aspects of the research are omitted except in cases where their inclusion is necessary and/or related to the purpose of this article.

## Method

### Participants and procedure

The sampling frame (Särndal et al. 2003) comprised two groups of female college students from a university with an enrollment of approximately 14,000 in the upper Midwest of the USA. The first group (STEM “persisters”) consisted of female students who were enrolled in STEM programs^2^ for the spring 2016 semester who had completed at least one semester in a STEM program or remained a STEM major at the time of the study. The second group (STEM “switchers”) included female students who were enrolled in non-STEM programs^3^ (e.g., all academic fields that are not considered STEM) during spring 2016 semester, but who at one time had been enrolled in STEM programs for at least one semester. Students who switched between STEM programs were also referred to as STEM switchers. The school e-mail addresses of 2055 female students (including STEM switchers and STEM persisters) were randomly chosen by the institution’s office of information research, which then invited students to participate in the online survey. Of the 392 who responded to the invitation, 356 started and finished the survey, yielding a final response rate of 17.32%. One reason that this response rate may have been low when compared to the average online survey response rate of about 25% (FluidSurveys Team 2014) is that the online survey was administered in the summer, when most students were on break. Thus, it is possible that some students were not checking their school e-mail accounts within the time frame the survey was administered and, therefore, did not respond to the survey. As Comrey and Lee (1992) have noted, a relatively small sample size could affect the reliability of survey instruments. Comrey and Lee propose the following benchmark as a guide to determining the adequacy of total sample size for factor analysis in a research study: 50 = very poor, 100 = poor, 200 = fair, 300 = good, 500 = very good, and 1000 or more = excellent (p. 217). Thus, the sample size of 356 participants for the current study is *good*.

The time frame for recruitment was driven by program requirements for the primary researcher, whose visa required completion of graduate studies by the following year. IRB approval was granted just before the summer term, and it was determined that data collection should begin and, if needed, be supplemented by additional data collection in the fall term. Initial analysis suggested that the response pool from the summer term was representative and sufficient to proceed with analyses without further data collection.

All female students who participated in this study were full-time undergraduate and graduate students. About 76% of the participants were enrolled in bachelor’s degree programs, while the rest were enrolled in master’s (13%) and doctoral (11%) degree programs. Of the total number of undergraduate students who participated in the study, 33.33% were first-year students (i.e., first year, second semester), 28.15% were second-year students, 25.92% were third-year students, and 12.59% were in their senior year. With regard to the total number of graduate student participants, the majority of them (38%) were first-year students (i.e., first year, second semester). About 92% of the participants identified themselves as U.S. citizens or permanent residents. The average age of the participants was 23.36 years (SD = 5.84). The data analysis also revealed that 297 of the total number of participants were STEM persisters, while 59 of them were STEM switchers. Participants who completed the survey and also agreed on the survey to have their e-mail addresses entered into a random drawing had the chance to win a $50 Amazon.com gift card.

Because of privacy policies at the university, it was not possible to ascertain whether the characteristics of those who chose not to participate differed from those who chose to participate. Because the researchers wanted to focus exclusively on women for this study, the Office of Institutional Research was required to draw the sample and to send the recruitment e-mail out to the students; it was up to those students to self-select and identify during the survey.

### Measures

To measure the study participants’ attitudes toward STEM education and careers, we adapted an instrument from an initial study developed to assess college students’ attitudes toward computer technology (see Appianing and Eck 2015). That instrument, which was called VIES and was also based on Eccles’ expectancy-value model, consisted of three subscales made up of 22 Likert-type scale items (1 = “Strongly Disagree” to 5 = “Strongly Agree”). The VIES was developed to measure college students’ attitudes and beliefs about computer technology majors, and the three subscales on the VIES measure the value that students place on computer technology fields, their interest in pursuing a degree in computer technology, and their expectations for success in computer technology fields.

For the current study, in order to capture participants’ perceptions regarding STEM education and careers specifically, each of the 22 VIES subscale items was adapted to include the word “STEM,” and all 22 items from the three subscales were presented without categorization (i.e., they were intermingled regardless of subscale). While the VIES instrument had been previously validated during the initial study, the modification of items for the current study (in order to focus the responses on STEM rather than computing technology) required that an additional confirmatory factor analysis be conducted.

## Results

Prior to factor analysis, we performed descriptive statistics (see Table 6 in Appendix 1) using SPSS version 24.0 (SPSS Inc., Chicago, IL, USA) in order to identify missing data and outliers in the VESAS survey data. The descriptive statistics showed no missing data on the VESAS subscales. Outliers were determined by calculating and examining the z-scores for the individual items on the VESAS. Research suggests that z-scores that fall within the range of + 3.29 and − 3.29 are not considered outliers (Field 2009; Tabachnick and Fidell 2007). Because no z-scores fell outside this range, it was determined that there were no outliers in the VESAS survey data. All negatively worded items (e.g., “I dislike STEM courses” and “Working in a STEM field would be a waste of my time”) were reverse-scored prior to analysis. This was done to improve interpretability by ensuring that high scores on the VESAS subscales reflect high levels of the attributes being measured (e.g., perceived value of STEM fields). For instance, on our 5-point Likert-type scale items, if a participant responded, Strongly Disagree (where Strongly Disagree = 1) to the statement “I dislike STEM courses,” the participant’s response was recoded to a value of 5 in order to reflect the high level of perceived value of STEM fields indicated by the participant’s response. Put another way, reverse scoring a VESAS item is based on the assumption that a participant who strongly disagrees with the statement “I dislike STEM courses” is in effect saying that she likes STEM courses, and therefore places a high value on STEM fields. A standard convention level of *p* < 0.05 was used for evaluating statistical significance of all the quantitative analyses performed in this study.

### Factor analysis

We performed a confirmatory factor analysis using SPSS to test for the construct validity of the VESAS subscale items. While the literature indicated differing opinions regarding the sample size required to perform a factor analysis of scale items to which participants have responded, a number of studies (e.g., MacCallum et al. 1999; Pett and Lackey 2003; Tabachnick and Fidell 2007; Williams et al. 2010) cite the work of Comrey and Lee (1992). As referenced earlier, Comrey and Lee’s guidelines suggest that the sample size for the current study (356) was good and thus sufficient for factor analysis.

#### Kaiser-Meyer-Olkin and Bartlett’s test of sphericity

Apart from reviewing literature, statistical tests were also performed in SPSS to check whether the sample size for the current study was adequate for factor analysis. These tests included the Kaiser-Meyer-Olkin (KMO) test, which is a measure of sampling adequacy, and Bartlett’s test of sphericity, which tests the null hypothesis that the correlation matrix is an identity matrix (Hagemeier and Murawski 2014). The value of the KMO can range between 0 and 1. The sample size is said to be adequate for factor analysis if the value of KMO is greater than .50, and Bartlett’s test of sphericity result is statistically significant, that is, *p* < 0.05 (Field 2009; Williams et al. 2010). For the current survey data, the value of KMO was .96, indicating that the sample size was adequate for factor analysis. Furthermore, the significance level of Bartlett’s test of sphericity was less than 0.01, indicating the absence of an identity matrix. Thus, the results of the KMO and Bartlett’s test of sphericity suggested that the sample size in relation to the number of items of the VESAS subscales was appropriate for factor analysis.

#### Factor extraction

The current study used the principal component analysis to test the underlying factor structure of the VESAS subscale items. Several criteria were used in the process of determining the number of factors to extract, including Kaiser’s rule (eigenvalues > 1), the Scree Plot test, and the cumulative percent of variance extracted. This approach to selecting factor items is recommended by several studies (e.g., Costello and Osborne 2005; Field 2009; Williams et al. 2010).

To determine the number of factors to extract, all 22 VESAS subscale items were included in the factor analysis. The first factor analysis test was performed with the following specifications: all rotated factor loadings below .10 were suppressed; the oblique rotation method (direct oblimin) with factor extraction via eigenvalues greater than 1 was used. The oblique rotation method was used because the results of the initial study suggested that the VESAS subscales were correlated.

Results from the initial factor analysis test produced three factors from the principal component analysis with eigenvalues greater than 1.0, suggesting a three-factor structure (see Table 7 in Appendix 2 for the factor loadings from the first factor analysis). The three factors extracted accounted for 67.33% of the variance. Commonalities were generally greater than or close to .70, but the pattern matrix displayed several weak and/or cross-loading items on all the factors. According to Costello and Osborne (2005), any survey item that loads at .32 or higher on two or more factors is a cross-loading item. Further, the loadings on the third factor were generally below .32, which is the minimum loading value recommended by Costello and Osborne. Finally, the Scree Plot test for the initial factor analysis suggested a two-factor structure. Therefore, a second factor analysis was conducted with the decision to extract only two factors, including suppressing rotated factor loadings below .40 in order to eliminate weak and cross-loading items.

The pattern matrix table for the second factor analysis revealed two distinct factors, with 14 items loading on the first factor and 8 on the second factor (see Table 8 in Appendix 3 for the loadings from the second factor analysis). The two factors extracted accounted for 62.29% of the variance. The factor loadings were generally .60 or higher. One possible reason why the principal component analysis extracted a two-factor solution (i.e., value-interest and expectations for success) for the current student sample as compared to the three-factor solution (value, interest, and expectations for success) suggested by our initial study may be due to the disparity in sample sizes (sampling error) between the current study and the initial study. As pointed out earlier, factor loadings produced from larger samples tend to show “smaller standard deviations of loadings across repeated samples” (MacCallum et al. 1999, p. 85). The result revealed by the current factor analysis also suggested that the students did not differentiate between value and interest, which also supports the Eccles et al. (1983) expectancy-value model that defines the construct of interest as a component of the subjective task value.

All the 14 items that loaded on factor 1 were summed into one scale, while the eight items that loaded on factor 2 were also summed into another scale. We carefully examined descriptive statistics (see Table 1) including skewness and kurtosis scores in order to identify items that appeared to be problematic. Results of the descriptive statistics indicated that the individual items and summed-scale distributions all approached normality based on the statistical values of skewness and kurtosis. For the summed scales, the skewness and kurtosis values were between the acceptable ranges of ± 1.0, whereas the individual items were between the acceptable ranges of ± 2.0, which is also considered acceptable (George and Mallery 2010; Tabachnick and Fidell 2013).

**Table 1:** Final rotated factor loadings, reliability coefficients, means, standard deviations, skewness, and kurtosis

Item no.	Item wording	Factor loadings		*M*	SD	SK	*K*
		1	2				
Perceived value of STEM fields (*α* = .90)							
Item 3	I find STEM-related jobs very interesting	.91	–	4.24	.82	− 1.13	1.51
Item 2	I would take a course in STEM even if it were not required	.86	–	3.98	.95	− 1.02	.97
Item 5	STEM is an important field for me	.78	–	4.27	.77	− .95	.81
Item 14	I dislike STEM courses (R)	.73	–	4.03	.87	− 1.00	1.10
Item 9	STEM is a good college major for me	.72	–	4.00	1.01	− .91	.22
Item 4	I don’t think working in a STEM field would help me achieve my professional aspirations (R)	.61	–	4.15	1.03	− 1.10	.29
Item 19	I feel I would have something to be proud of as a STEM professional	.59	–	4.33	.74	− 1.19	2.16
Item 1	Working in a STEM field would be a waste of my time (R)	.51	–	4.54	.75	− 1.81	3.50
Expectations for success in STEM careers (*α* = .89)							
Item 20	I don’t think I will succeed in a STEM field (R)	–	.88	4.07	.95	− 1.12	1.10
Item 18	I don’t think I can make an impact if I take on a STEM-related job (R)	–	.85	4.07	.93	1.06	.88
Item 16	I would certainly feel useless in a STEM-related job (R)	–	.73	4.19	.87	− 1.19	1.64
Item 15	I feel I have what it takes to succeed in a STEM-related job	–	.70	4.07	.86	− .81	.44
Item 21	I would be able to succeed in a STEM field as well as most other people	–	.68	4.03	.87	− .96	1.05
Item 22	I do not think I can achieve anything meaningful as a STEM professional (R)	–	.66	4.26	.83	− 1.11	1.14
Item 17	I feel I have a number of good qualities to be successful in a STEM field	–	.65	4.17	.74	− .99	2.18
Eigenvalues		8.09	1.14				
Percentage variance (%)		53.92	7.57				

Total variance explained by the two factors = 61.49%. “R” indicates that item was reverse-coded prior to analysis

*M* mean, *SD* standard deviation, *SK* skewness, *K* kurtosis

##### Internal consistency tests

To determine the internal consistency of the two factors extracted, Cronbach’s alpha coefficients were calculated. The reliability coefficient for the first factor was .95, while that of the second factor was .90. Various literature sources indicated two main schools of thoughts as to what values for alpha are acceptable. The first school of thought is that values for alpha ranging between .70 and .95 are acceptable (e.g., Bland and Altman 1997; George and Mallery 2005). The second school of thought holds that values for alpha between .70 and .90 are acceptable (e.g., Streiner 2003; Tavakol and Dennick 2011). While acknowledging the differing views regarding acceptable values of alpha, the current study adopted the second school of thought. This is because researchers within this school of thought made a strong case as to why the acceptable values of alpha should not exceed .90, maintaining that an alpha value greater than .90 may suggest redundancy.

In other words, the scale items may be asking the same question in many different ways. Since Cronbach’s alpha for factor 1 exceeded the .90 ceiling, all of the 14 items that loaded on factor 1 were reexamined statistically for redundancy.

To examine factor 1 items further, we conducted reliability analyses using SPSS by including inter-item correlations (IICs) and examined changes in alpha levels if each scale item was deleted. Results indicated that the reliability of factor 1 subscale would be strengthened if item 7 was removed. Additionally, as the initial Cronbach’s alpha was very high for factor 1 subscale (.95), IICs were examined for items which may be too similar. Items 6, 7, 8, 10, 11, 12, and 13 (see Table 2), all had IIC coefficients between .75 and .80. While the literature suggests .80 as a cutoff of IICs, the analysis showed that removal of these items would still produce a strong subscale. Accordingly, these items were removed along with item 7 (Table 2).

**Table 2:** Items that were removed from the VESAS subscales and their rationale for doing so

Items removed from the VESAS subscales	Reason(s) for removal of item
Item 11 (Being in a STEM class would be fun for me)	These 4 items were too similar to item 2 (I would take a course in STEM even if it were not required) and item 3 (I find STEM-related jobs very interesting)
Item 12 (The idea of being in a STEM class excites me)	
Item 13 (I would enjoy taking STEM courses)	
Item 6 (I would enjoy working in a STEM field)	
Item 8 (I am not interested in a degree in STEM)	These 2 items were too similar to item 14 (I dislike STEM courses)
Item 10 (STEM classes are boring)	
Item 7 (I would rather do something else than take on a STEM-related job)	This item was removed to improve the internal consistency of the scale

Following the removal of the seven items from the VIES subscales, a third factor analysis was performed with the remaining 15 items by utilizing the same specifications used during the second factor analysis. As expected, the principal component analysis extracted two factors, with both the Scree Plot test and the total variance explained table in SPSS confirming a two-factor structure. The total variance explained by the two factors extracted was 61.49%.

Commonalities were generally between .50 and .75. Eight items loaded on factor 1, while seven items loaded on factor 2, with factor loadings ranging from .51 to .91. Table 1 shows the final rotated factor loadings, reliability coefficients, means, standard deviations, skewness, and kurtosis for the modified VIES subscales.

#### Content of the final 15 items retained

After carefully examining the final rotated factor loadings, we confirmed that the eight items that loaded on factor 1 reflected the original VIES “value” subscale. Therefore, factor 1 was named “Perceived Value of STEM Fields.” The perceived value of STEM fields subscale was used to assess the degree to which the participant valued STEM majors and careers. It was determined that the perceived value of STEM fields subscale items reflected the four key components of the subjective task value identified by Eccles et al. (1983). For example, item 2, “I would take a course in STEM even if it were not required,” reflects intrinsic value; item 19, “I feel I would have something to be proud of as a STEM professional,” reflects the attainment value; item 9, “STEM is a good college major for me,” echoes the utility value; and item 1, “Working in a STEM field would be a waste of my time,” mirrors the cost value (see Table 3).

**Table 3:** Perceived value of STEM fields subscale items and their subjective task value match

Item no.	Item wording	Subjective task value component
2	I would take a course in STEM even if it were not required	Intrinsic value
19	I feel I would have something to be proud of as a STEM professional	Attainment value
9	STEM is a good college major for me	Utility value
1	Working in a STEM field would be a waste of my time	Cost value

The seven items that loaded on factor 2 paralleled the original VIES “expectations for success” subscale. Therefore, factor 2 was named “Expectations for Success in STEM Careers.” The expectations for success in STEM careers was used to assess the degree to which participants expected to do well in STEM-related jobs. The expectations for success in STEM Careers subscale also reflected the expectation for success construct in Wigfield and Eccles’s (1992) expectancy-value model.

##### Test of normality and reliability test of the final 15 items retained

All of the items that assessed perceived value of STEM fields were summed into one scale. Higher scores meant participants valued STEM fields and careers more. Items measuring expectations for success in STEM careers were also summed into one scale. Higher scores meant higher expectations for success in STEM-related jobs. In order to determine whether the survey data follow a normal distribution, descriptive statistics were calculated and examined for both individual items and summed-scale distributions. Results of the descriptive statistics indicated that the individual items (Table 1) and summed-scale distributions (Table 4) all approached normality based on the statistical values of skewness and kurtosis.

**Table 4:** Summed-scale distributions for VESAS

Analysis	Perceived value of STEM fields	Expectations for success in STEM careers
Mean	33.53	28.85
Standard deviation	5.43	4.71
Skewness	− .87	− .58
Kurtosis	.38	− .17

For the summed scales, the skewness and kurtosis values were within the acceptable range of ± 1.0, whereas the range for the individual items was between the acceptable ranges of ± 2.0. However, the kurtosis value for item 1 (see Table 1) exceeded the + 2 threshold, which was an indication that the item could be problematic. This abnormality may have been the result of the fact that a disproportionately high number of participants (90%) indicated that working in a STEM field would not be a waste of their time. Such a peaked frequency distribution was expected since a majority of the participants were STEM persisters, while STEM switchers who probably felt that working in a STEM field would be a waste of their time were few. Indeed, the data analysis (Table 1) showed that item 1 had the lowest factor loading of .51, which means that it contributed relatively less to the perceived value of STEM fields subscale. However, according to Costello and Osborne (2005), a scale item with a factor loading of .50 or higher is a good item. In addition, item 1 was the only perceived value of the STEM fields scale item that assessed cost value. Therefore, item 1 was retained for further analysis.

To determine the internal consistencies (or reliabilities) of the perceived value of STEM fields and expectations for success in STEM careers subscale items, Cronbach’s alpha coefficients were calculated. The reliability coefficient for the perceived value of STEM fields subscale was .90, while that of the expectations for success in STEM careers subscale was .89 (see Table 1). These results suggested that both the perceived value of STEM fields and expectations for success in STEM careers subscale items had sufficient internal consistency and could thus be used for assessing variables of interest in the current study.

### Correlation between the expectancy-value model constructs

We expected that the VESAS subscales would be somewhat related if they are, in fact, measuring the aspects of a common trait (i.e., motivational belief). Thus, a bivariate correlation (Cronk 2012) was calculated to determine the relationship between the subscales of the VESAS. We found a strong, positive significant correlation between perceived value of STEM fields and expectations for success in STEM careers (*r* = .76, *p* < 0.001). This strong, positive correlation may be of particular interest to achievement motivation theorists, including Eccles et al. (1983), who believe that individuals’ educational and vocational choices are influenced by both their subjective values and expectations for success.

### Group differences between STEM switchers and STEM persisters on the VESAS subscales

Assuming the validity of Eccles et al.’s (1983) theory of values and expectations, an instrument such as the VESAS which is designed to measure those factors should be able to show that people with different levels of values and expectations will behave in a manner consistent with those levels. Accordingly, group means on the VESAS were used to examine differences between women STEM switchers and women STEM persisters. The analyses revealed statistically significant differences in means between women STEM persisters (*M* = 34.81, SD = 4.52) and women STEM switchers (*M* = 29.64, SD = 4.27) on the perceived value of STEM fields subscale. Cohens’^4^ (1988) effect size value suggested a very large practical significance (*d* = 1.55). Similarly, we found statistically significant differences in means between women STEM persisters (*M* = 29.64, SD = 4.27) and women STEM switchers (*M* = 25.02, SD = 4.94) on the expectations for success in STEM careers subscale. Cohen’s effect size value suggested a very large practical significance (Cohen *d* = 1.00). These results provide further evidence that the VESAS produces results in the direction predicted by Eccles et al.’s theory.

## Discussion

We developed the VESAS as an instrument for measuring college students’ attitudes toward STEM programs and careers. Given that women are underrepresented in the STEM fields and careers (Chen 2013; Griffith 2010) and the fact that the STEM literature indicates that female students are more likely to switch from STEM programs than their male counterparts (Chen 2015; Hill et al. 2010), it is imperative to assess female college students’ attitudes toward STEM. Furthermore, few, if any, studies have measured female STEM persisters and female STEM switchers’ attitudes toward STEM using Eccles et al.’s (1983) expectancy-value model.

Our analysis indicated that the VESAS is psychometrically sound in terms of construct validity and internal consistency. The analysis revealed the existence of two independent constructs (factors) that relate to the study participants’ expectations for success in STEM careers and perceived value of STEM fields with high Cronbach’s alphas. Our analysis, therefore, suggests that the two-factor structure of the VESAS supports Eccles et al.’s (1983) expectancy-value model.

The two-factor structure was anticipated because we believe, and the expectancy-value model holds (Eccles et al. 1983), that achievement-related tasks (i.e., the decision to choose and persist in STEM majors) may be influenced by the value that the individuals attach to STEM fields and how well they expect to perform in STEM-related careers. Thus, it was not surprising that the result of the current study indicated that women who persisted in STEM majors had higher expectations for success in STEM careers and placed a higher value on the STEM fields than did women who switched from STEM majors.

The study also revealed that the expectations for success that the participants held about STEM careers were significantly related to the value that they attached to the STEM fields. This strong relationship between perceived value and expectancy implies that, all things being equal, students with low expectations for success in STEM careers may have low perceived value for STEM fields. In other words, students who do not expect to do well as would-be chemical engineers, for example, may not show much interest in chemical engineering-related classes, because those classes have neither attainment value nor utility value for them. Thus, these students would view taking chemical engineering classes as a waste of time (cost value). As noted by Plunkett et al. (2010), expectation for success for a future task is influenced by the success achieved by the individual in a previous related task. Therefore, the chemical engineering students with low expectations for success in STEM careers may have concluded from their poor performance in STEM classes that such performance was an indication of their inability to do well in a chemical engineering career.

### Implications

A full account of the ways the VESAS can be used to inform efforts to attract and retain women to STEM majors is beyond the scope of this article. We will provide a more in-depth analysis based on the study population referred to in this article in a future publication. For now, suffice it to say that the underrepresentation of women in STEM fields is, in part, driven by the number of women who seek and persist in STEM majors in college and that the reasons they may choose to seek non-STEM majors and/or switch out of STEM is an important factor in developing interventions to recruit and retain them. The VESAS appears to be a valuable potential tool for achieving this. To illustrate this, consider that the likelihood of persisting through graduation in a STEM major may be driven by the interaction of ability (the cognitive skills required by the STEM field and its prerequisite subjects) and the two constructs measured by the VESAS. Table 5 presents all possible interactions of these factors.

**Table 5:** All possible interactions of ability, perceived value of STEM fields, and expectations for success in STEM careers

Student	Ability	Value		Expectations	
		High	Low	High	Low
1	Sufficient	X		X	
2	Sufficient	X			X
3	Sufficient		X	X	
4	Sufficient		X		X
5	Insufficient	X		X	
6	Insufficient	X			X
7	Insufficient		X	X	
8	Insufficient		X		X

Ability is beyond the scope of this instrument. If students are early in their educational experience (precollege), educational interventions such as tutoring and high-quality STEM class instruction may be effective interventions to remediate for ability, thus increasing the likelihood of recruiting and retaining more students. If a student is in college, there may not be time to address ability in the traditional 5-year college plan and its attendant prerequisite courses. If ability is not sufficient, it is likely that a student would be counseled out of STEM majors (lack of success will lead to low expectations for success and thus to switching majors later in any case). There is no evidence that women are innately less able to be successful in STEM areas, and evidence suggests that the underrepresentation of women in STEM is unrelated to ability (e.g., Astorne-Figari and Speer 2017; Cheryan et al. 2016; Martin 2015).

However, if we assume that students have (or will have by college) sufficient ability (students 1–4 in Table 5), the VESAS may be valuable in identifying key students who, with the right support structures, role models, or high-quality STEM curriculum, may become persisters.

Various social phenomena (e.g., lack of role models, portrayal of STEM as a male-dominated field in popular culture, and biased expectations on the part of teachers and parents) may disadvantage women in the areas of value and expectation for success regarding STEM, thus leading in part to their underrepresentation. One might hypothesize that a significant portion of women who fail to seek or who drop out of STEM majors and fields comprise students with sufficient ability but low value (student 3), low expectations for success (student 2), or both (student 4). Student 1 needs no intervention—one can easily imagine that a high proportion of STEM graduates are in this classification. Student 2, on the other hand, may be a key constituency for increasing the likelihood of selecting STEM majors and being a STEM persister. One can imagine many women, for example, who have low expectations for success because of the culture of STEM (in popular media, in STEM classes) and the lack of role models or support structures (e.g., first-generation college students, no friends who are STEM majors), either do not choose or switch out of STEM majors. Student 3 has the ability and expects to succeed but does not have a high value for STEM and is less likely to find long-term professional fulfillment. Student 3 might benefit from considering other STEM majors he or she would value or, with the right instructional intervention, might come to see more value in STEM applications beyond the classroom. Student 4 probably would be counseled out of STEM majors as well, unless he or she has simply not been exposed to the right kinds of instructional experiences and if there are time and resources to do so.

Taken together, the results of this study suggest that the VESAS can be used as a tool to measure the expectancy and value beliefs among female college students. For example, the VESAS can be administered to students prior to or during their first year in STEM programs in order to see how their scores on the expectations for success in STEM careers and perceived value in STEM fields subscales change over time and to better understand when interventions might be needed and with whom. Thus, the purpose for administering VESAS is to identify students with low self-efficacy (Bandura 1977) and subjective task value (Eccles 2005) beliefs and to offer support and encouragement to them before they make the decision to switch from collegiate STEM programs.

### Limitations

The VESAS, like all quantitative surveys, is based on self-assessments rather than objective measures (Debois 2016; Wyse 2012). Therefore, the results of the current study should be interpreted with caution. Because the sample population was drawn from a single institution and because the response rate for the survey was relatively low, future research should include larger and more diverse sample populations. While the expectancy-value model has been studied with diverse populations and should therefore be equally valid with men and women, the sample for this study was exclusively female. Thus, future studies should validate the VESAS with males.

## Conclusions and future work

The VESAS is a valid instrument for measuring the values and expectations female college STEM majors have toward STEM. Researchers and college personnel who are interested in using Eccles et al.’s (1983) expectancy-value theory to help understand who will seek and persist in STEM majors and to use those results to inform recruiting and intervention programs can adopt the VESAS for those purposes.

## Appendix 1

**Table 6:** Descriptive statistics for the individual VESAS items (*N* = 356)

Item no.	Wording	*M*	SD	SK	*K*
Item 1	Working in a STEM field would be a waste of my time (R)	4.54	.75	− 1.81	3.5
Item 2	I would take a course in STEM even if it were not required	3.98	.95	− 1.02	.97
Item 3	I find STEM-related jobs very interesting	4.24	.82	− 1.13	1.51
Item 4	I don’t think working in a STEM field would help me achieve my professional aspirations (R)	4.15	1.03	− 1.10	.289
Item 5	STEM is an important field for me	4.27	.77	− .95	.81
Item 6	I would enjoy working in a STEM field	4.18	.91	− 1.22	1.38
Item 7	I would rather do something else than take on a STEM-related job	3.77	1.04	− .59	− .30
Item 8	I am not interested in a degree in STEM	4.10	1.06	− 1.78	.74
Item 9	STEM is a good college major for me	4.00	1.01	− .91	.22
Item 10	STEM classes are boring	3.95	.86	− .60	.09
Item 11	Being in a STEM class would be fun for me	3.94	.89	− .92	1.02
Item 12	The idea of being in a STEM class excites me	3.85	.94	− .63	.15
Item 13	I would enjoy taking STEM courses	4.00	.87	− 1.02	1.42
Item 14	I dislike STEM courses (R)	4.03	.87	− 1.00	1.10
Item 15	I feel I have what it takes to succeed in a STEM-related job	4.07	.86	− .81	.44
Item 16	I would certainly feel useless in a STEM-related job (R)	4.19	.87	− 1.19	1.64
Item 17	I feel I have a number of good qualities to be successful in a STEM field	4.17	.74	− .99	2.18
Item 18	I don’t think I can make an impact if I take on a STEM-related job (R)	4.07	.93	1.06	.88
Item 19	I feel I would have something to be proud of as a STEM professional	4.33	.74	− 1.19	2.16
Item 20	I don’t think I will succeed in a STEM field (R)	4.07	.95	− 1.12	1.10
Item 21	I would be able to succeed in a STEM field as well as most other people	4.03	.87	− .96	1.05
Item 22	I do not think I can achieve anything meaningful as a STEM professional (R)	4.26	.83	− 1.11	1.14

“R” indicates that item was reverse-coded prior to analysis

*M* mean, *SD* standard deviation, *SK* skewness, *K* kurtosis

## Appendix 2

**Table 7:** Rotated factor loadings from the first factor analysis

Item no.	Wording	Factor loadings		
		1	2	3
Item 13	I would enjoy taking STEM courses	.976	− .151	− .117
Item 12	The idea of being in a STEM class excites me	.952	− .132	
Item 11	Being in a STEM class would be fun for me	.936	− .133	− .107
Item 3	I find STEM-related jobs very interesting	.773		− .141
Item 10	STEM classes are boring	.725	.109	.208
Item 2	I would take a course in STEM even if it were not required	.712		
Item 14	I dislike STEM courses (R)	.687	.269	.239
Item 6	I would enjoy working in a STEM field	.677	.186	− .127
Item 9	STEM is a good college major for me	.660	.209	− .139
Item 5	STEM is an important field for me	.624	.129	− .198
Item 8	I am not interested in a degree in STEM	.623	.296	.155
Item 7	I would rather do something else than take on a STEM-related job	.513	.406	.180
Item 19	I feel I would have something to be proud of as a STEM professional	.463	.198	− .320
Item 10	STEM classes are boring	− .123	.810	− .145
Item 22	I do not think I can achieve anything meaningful as a STEM professional (R)		.804	
Item 16	I would certainly feel useless in a STEM-related job (R)		.776	− .107
Item 20	I don’t think I will succeed in a STEM field (R)		.765	− .178
Item 1	Working in a STEM field would be a waste of my time (R)	.222	.629	.122
Item 4	I don’t think working in a STEM field would help me achieve my professional aspirations (R)	.352	.561	.180
Item 21	I would be able to succeed in a STEM field as well as most other people	.142	.243	− .607
Item 17	I feel I have a number of good qualities to be successful in a STEM field	.267	.219	− .607
Item 15	I feel I have what it takes to succeed in a STEM-related job	.300	.280	− .528

## Appendix 3

**Table 8:** Rotated factor loadings (second factor analysis), means, and standard deviations

Item no.	Wording	Factor loadings		*M*	SD
		1	2		
Item 12	The idea of being in a STEM class excites me	.969	–	3.85	.94
Item 13	I would enjoy taking STEM courses	.968	–	4.00	.87
Item 11	Being in a STEM class would be fun for me	.930	–	3.94	.89
Item 10	STEM classes are boring	.797	–	3.95	.86
Item 14	I dislike STEM courses (R)	.772	–	4.03	.87
Item 3	I find STEM-related jobs very interesting	.761	–	4.24	.82
Item 2	I would take a course in STEM even if it were not required	.713	–	3.98	.95
Item 8	I am not interested in a degree in STEM	.687	–	4.10	1.06
Item 6	I would enjoy working in a STEM field	.672	–	4.18	.91
Item 9	STEM is a good college major for me	.652	–	4.00	1.01
Item 5	STEM is an important field for me	.598	–	4.27	.77
Item 7	I would rather do something else than take on a STEM-related job	.584	–	3.77	1.04
Item 4	I don’t think working in a STEM field would help me achieve my professional aspirations (R)	.425	–	4.15	1.03
Item 19	I feel I would have something to be proud of as a STEM professional	.406	–	4.33	.74
Item 18	I don’t think I can make an impact if I take on a STEM-related job (R)	–	.858	4.07	.93
Item 20	I don’t think I will succeed in a STEM field (R)	–	.836	4.07	.95
Item 16	I would certainly feel useless in a STEM-related job (R)	–	.792	4.19	.87
Item 22	I do not think I can achieve anything meaningful as a STEM professional (R)	–	.774	4.26	.83
Item 21	I would be able to succeed in a STEM field as well as most other people	–	.663	4.03	.87
Item 17	I feel I have a number of good qualities to be successful in a STEM field	–	.635	4.17	.74
Item 15	I feel I have what it takes to succeed in a STEM-related job	–	.633	4.07	.86
Item 1	Working in a STEM field would be a waste of my time (R)	–	.481	4.54	.75

## Footnotes

1 First-generation students are defined as those who are the first members of their families to go to college.

2 In this study, the following academic fields are designated as STEM programs/majors: Air Traffic Control, Airport Management, Applied Economics, Atmospheric Sciences, Aviation, Aviation Management, Biology, Biomedical Sciences, Chemical Engineering, Chemistry, Civil Engineering, Commercial Aviation, Communication Science and Disorders, Computer Science, Clinical Psychology, Earth System Science and Policy, Electrical Engineering, Energy Engineering, Energy Systems Engineering, Engineering, Environmental Engineering, Environmental Geoscience, Forensic Psychology, Forensic Science, Geography, Geological Engineering, Geology, Geographic Information Science, Graphic Design Technology, Industrial Technology, Information Systems, Applied Economics, Mathematics, Mechanical Engineering, Medicine, Nursing, Occupational Therapy, Petroleum Engineering, Physics, Unmanned Aircraft System Operator, Psychology, Space Studies, and Technology.

3 Examples of non-STEM majors include Anthropology, Business, Education, Communications, Counseling, Criminal Justice, English, Human Resources, Law, Management, Public Administration, Social Work, Sociology, and MBA (Source: Office of Institutional Research, a Midwestern Public University, 2016).

4 Cohen (1988) offered a rough rating scale for determining effect sizes: small (*d* = 0.2), medium (*d* = 0.5), and large (*d* = 0.8).
